# Recreation in Relation to the Health of the People

**Published:** 1891-01-31

**Authors:** 


### Recreation in Relation to the Health of the People.

### II.—BEGINNING AT THE BOTTOM.

We began our first article by asking the question: What is health? Let us follow suit, and begin our second by enquiring: What is recreation? If this question were asked of various persons, and in various circles, probably no two answers would be alike, so much are men's ideas on the subject guided by their personal tastes, as well as by the circumstances of their daily life. To one, recreation means the long country walk for which he so often sighs in vain; to another it means diving deep into some abstruse treatise which to somebody else is part of the distasteful paraphernalia of office-life. Mr. Gladstone, as we all know, looks upon an essay on Homer, or a happy half-hour with the axe, as *his* recreation; while to many a labourer or artisan no recreation seems worth mentioning but an evening spent in the public-house, with a pipe, and a tap warranted to get him duly "forrarder."

Still, all our readers will probably agree to this: that the term "recreation" includes anything and everything that gives rest to the tired mind or body, whichever it may chance to be,—that relieves the monotony of every-day life and labour,—that once more restores, or tends to restore, the natural balance, so to speak, of mind and body, of care-fulness and light-heartedness, which the toils and anxieties of the day often cause us to lose, and which, alas! the life that too many of us lead seems sometimes to altogether destroy. Thus it is only natural that recreation should present itself to us in many different aspects, varying not only with the lives which we lead, but with our personal idiosyncrasies, and still more, with the degree of cultivation which we possess. Those who wish to supply recreation to the people, then, must walk warily, and must not hastily conclude that because a certain type of recreation—say, a moderate amount of brain exertion—seems specially appropriate to a man who spends all his working-day in some one of the mechanical tasks to which the excessive subdivision of labour has condemned him, he will at once be ready to grasp at it. That he should desire it, is much to be wished, and much to be aimed at; but he will have to be educated up to it first, and as a preliminary it will be best (we are talking of him, of course, not as an individual, but as a specimen of a class) to work upon such tastes as he already possesses, doing what we can to recreate and educate him by means of them, and at the same time gaining a hold upon him which may in time lead to higher things. If the palate has to be educated up to a due appreciation of olives, no one needs to be taught to like sugar; and in the same way, there are certain innocent recreations which all can appreciate, or if there are any exceptions, they but prove the rule. To find a person who does not care for music, for instance, is difficult enough; to find a *class* which does not care for it is impossible. Again, if we are catering for the young, the "rough-and-tumble" element is sure to take; witness the delight with which lads of all classes will watch the antics and pranks of Harlequin and Pantaloon. What is the exact meaning of a "knock-about comedian" we cannot say, but the title is evidently an attractive one, or comedians would not advertise themselves as such. "The more tumbling about there is, the better my Saturday night audiences like it," says the experienced lady-manager of the "Victoria Hall"; and as one glances at the eager and delighted faces in the gallery, one understands that the boisterous spirits must have a vent somehow, and that to see a "good old row" on the stage is the next best thing to letting off the steam *in propriâ personâ*. Gymnastics, again, are sure to be popular amongst boys, just because they also afford this chance of letting off the steam; and we quite agree with Mr. Edward Flower, Secretary of the Recreative Evening Schools Association, that it is often the wisest course to begin a night-school with nothing but gymnastics and other recreation. "The boys," says he, "do want to learn, but don't yet know it. They have to be caught with guile, to be inveigled into the search for knowledge. A teacher who has tact will soon find means of exciting a healthy curiosity, which is only another name for the thirst for knowledge."

Then, again, with regard to older people. Providing amusement for yourself—and perhaps for others too—is better, and probably in the end more pleasurable, than having all your amusement found for you. But it needs education (using the word in its wider meaning) to see this; and to begin one's work of providing recreation for the people by trying to inaugurate, say, a class for studying good music, would simply be to court defeat. Devote yourself first of all to giving good concerts now and then, which only ask of the people that they shall lend an ear; and seeds will thus be sown which will bear fruit later on, and very possibly result in a strong desire for the very classes that would once have excited no enthusiasm at all.

It is the same with intellectual pursuits. Popular lectures pave the way for classes and night schools; the man who has a little knowledge longs for more; he learns that there are more things in heaven and earth than are dreamt of in the philosophy of the little world around him; and there stir within him feelings of awe and wonder, and of eager desire to hear more of these things.

No better rule can be given to those who wish to raise the lives of the masses than this: *Begin at the bottom and work up.* And here we have before us three ways (doubtless there are more, but these are sufficient for our purpose) of getting at those who are at the bottom, so to speak: gymnastics for the young; entertainments, of a bright and varied character; and popular lectures. The first of these will be more conveniently dealt with under the head of Physical Recreation; for the present we will only consider the second and third, and mention very briefly some of the agencies for supplying the needs of the people in this respect.

The Royal Victoria Hall, in Waterloo Bridge Road, popularly known as "The Old Vic." is the best example we know both of "beginning at the bottom and working up," and of the success attending this plan. In 1880 the hall, which until then had been a disgrace to the name it bore, was taken on by a company, and opened as a Temperance Music Hall, variety entertainments being at first given every night in the week. It was the anxious desire of those interested in it to provide a counter-attraction—and an innocent counter-attraction—to the objectionable entertainments given at drink-selling halls; and in spite of many anxieties and various vicissitudes, the venture has proved a grand success, both with regard to the immediate results arrived at, and to the higher growths which have sprung from it.

We cannot but mention the indefatigable lady-manager, Miss Cons, who has freely given her services to it during the last ten years, and to whose pluck and perseverance so much of its success is due. "These ten years have turned my hair grey," she smilingly says; and, indeed, it is no wonder, when one hears of all her anxieties as to ways and means (we do not believe that a hall which has to compete with drink-selling music-halls can possibly pay its way, as things are at present), and thinks of the difficulties which beset one who is determined that the entertainment given shall be of an unobjectionable character, and yet wishes to attract a rough uncultured audience. Where draw the line between mere vulgarity and coarseness? It is not so easy sometimes—indeed, it is impossible, except for those who are up to the various devices of certain music-hall "artistes" for raising the nasty laugh which Miss Cons declares that she can always recognise—though fortunately she seldom, if ever, hears it now. She has met the difficulty by organising a committee of working-men, whose business it is at once to report to her anything objectionable, whether on the stage, or in the body of the room; and now that things are in full working order, it is the rarest thing for any singer to offend.

On one occasion, however, quite a dramatic little scene occurred. While a certain singer was amusing the company, a member of the committee hurried up with the information that in the first verse of the song something objectionable had been said or done. The singer was at once warned from the wings; but a minute later came the news—"He's done it again," and the "nasty laugh" was heard from the gallery. A stern "Stop the song, and come off the stage" greeted the singer's ears, but he declined to obey, and prepared to begin the third verse. Upon this the curtain was promptly rung down between him and the audience. There was a minute's silence, during which you might have heard a pin drop, and then a burst of applause.

Such a story speaks volumes, not only for the pluck and decision of the management, but for the success of the efforts which have been made to raise and purify the tastes of a really rough audience.

